# On variants and vaccines: The effectiveness of Covid-19 monoclonal antibody therapy during two distinct periods in the pandemic

**DOI:** 10.1371/journal.pone.0278394

**Published:** 2022-12-01

**Authors:** Vinay Srinivasan, Stacey E. Weinstein, Azra Bhimani, Nathan C. Clemons, Melissa Dinolfo, Christina S. Shin, Jacqueline Grier, Antonio Lopez, Jamia Braggs, Joni Boucher, Quanna N. Batiste, Omai B. Garner, Shangxin Yang, Tara Vijayan

**Affiliations:** 1 David Geffen School of Medicine, University of California, Los Angeles (UCLA), Los Angeles, California, United States of America; 2 Department of Medicine, David Geffen School of Medicine, UCLA, Los Angeles, California, United States of America; 3 Department of Pediatrics, David Geffen School of Medicine, UCLA, Los Angeles, California, United States of America; 4 Division of Infectious Diseases, David Geffen School of Medicine, UCLA, Los Angeles, California, United States of America; 5 Department of Pathology and Laboratory Medicine, David Geffen School of Medicine, UCLA, Los Angeles, California, United States of America; 6 Ambulatory and Community Practices, UCLA Health, Los Angeles, California, United States of America; 7 Department of Pharmaceutical Services, Ronald Reagan UCLA Medical Center, Los Angeles, California, United States of America; 8 Faculty Practice Group–Ambulatory Care Coordination, UCLA Health, Los Angeles, California, United States of America; 9 Department of Ambulatory Nursing, UCLA Health, Los Angeles, California, United States of America; 10 School of Nursing, UCLA, Los Angeles, California, United States of America; Stanford University School of Medicine, UNITED STATES

## Abstract

**Background:**

While Covid-19 monoclonal antibody therapies (Mab) have been available in the outpatient setting for over a year and a half, little is known about the impact of emerging variants and vaccinations on the effectiveness of Mab therapies. We sought to determine the effectiveness of Covid-19 Mab therapies during the first two waves of the pandemic in Los Angeles County and assess the impact of vaccines, variants, and other confounding factors.

**Methods and findings:**

We retrospectively examined records for 2209 patients of with confirmed positive molecular SARS-CoV2 test either referred for outpatient Mab therapy or receiving Mab treatment in the emergency department (ED) between December 2020 and 2021. Our primary outcome was the combined 30-day incidence of ED visit, hospitalization, or death following the date of referral. Additionally, SARS-CoV2 isolates of hospitalized patients receiving Mabs were sequenced. The primary outcome was significantly reduced with combination therapy compared to bamlanivimab or no treatment (aHR 0·60; 95% CI ·37, ·99), with greater benefit in unvaccinated, moderate-to-high-risk patients (aHR ·39; 95% CI ·20, ·77). Significant associations with the primary outcome included history of lung disease (HR 7·13; 95% CI 5·12, 9·95), immunocompromised state (HR 6·59; 95% CI 2·91–14·94), and high social vulnerability (HR 2·29, 95% CI 1·56–3·36). Two predominant variants were noted during the period of observation: the Epsilon variant and the Delta variant.

**Conclusions:**

Only select monoclonal antibody therapies significantly reduced ED visits, hospitalizations, and death due to COVID-19 during. Vaccination diminished effectiveness of Mabs. Variant data and vaccination status should be considered when assessing the benefit of novel COVID-19 treatments.

## Introduction

In November 2020, based on limited available data, the Food and Drug Administration issued Emergency Use Authorizations (EUA) for two monoclonal antibody (Mab) treatments for COVID-19: bamlanivimab, manufactured by Eli Lilly and casirivimab/imdevimab, manufactured by Regeneron [1, 2]. This issuance came in the wake of what was widely perceived as the third wave in the United States [3]. For California, the precipitous rise in cases than began in December 2020 was in fact the first major surge in the state.

The winter 2020 surge of COVID-19 placed immense strain on health systems across the country as cases, hospitalizations, and deaths skyrocketed to unprecedented highs [4]. ICUs rapidly reached capacity and remained full, and some hospitals in southern California started to consider rationing care to prioritize those who had the best chance of survival [4–6]. The surge was particularly devastating to high-risk and vulnerable populations, as overburdened hospitals struggled to provide quality care to all those in acute need [6]. In the absence of a widely available vaccine at that time, public health measures such as universal masking and social distancing remained the most effective ways to prevent infection and subsequent hospitalization. Effective vaccines were made available domestically through EUA as early as December 2020, but due to initial supply chain issues, there was a phased roll out, and select high-risk populations were prioritized in the early part of 2021. In addition, pervasive vaccine hesitancy limited large-scale uptake, and significant proportions of the population were susceptible to infection. The availability of outpatient therapeutics to prevent hospitalization in already-sick patients in the community was the only hope to reduce constraints on an overtaxed healthcare system.

The two Mabs initially approved for EUA directly target the spike protein required for viral entry and were demonstrated to reduce viral loads [7, 8]. In phase III trials, which typically consisted of just over 100 patients in each of the dosing and placebo arms, both bamlanivimab and casirivimab/imdevimab demonstrated modest efficacy in reducing hospitalizations and emergency department (ED) visits in recently diagnosed, high-risk outpatients with mild to moderate disease, particularly those who were considered high risk, and were tolerated well.

Given this, soon after the issuance of the EUA, the United States government agreed to purchase 1 million doses of bamlanivimab [9], and by early January to purchase all available doses of casirivimab/imdevimab [10] and offer it to healthcare systems at no cost. However, healthcare systems who chose to accept the doses had to create the infrastructure needed to administer the drug, requiring significant internal labor and costs. In late November 2020, UCLA Health developed an outpatient program to administer monoclonal antibody therapy. This program went live on December 10, 2020. Both available drugs were used on alternating days assuming clinical equipoise. Over the course of the year, bamlanivimab with etesevimab became available and was also used alternatively with casirivimab/imdevimab. Due to resource constraints, UCLA Health initially was only able to schedule infusions for two patients a day, outside of administration in the ED. After January 2, 2021, six infusion chairs per day were available for use and after January 20, 2021, a total of 12 infusion chairs per day were available for patients, five days a week. Although at the time we considered the drugs experimental, given that demand exceeded our ability to administer the drug, we created a rigorous point system to prioritize patients based on risk factors, as well as specific measures of socioeconomic vulnerability (S1 Table). The point system weighted BMI and older age the greatest given the marked benefit seen individuals with these risk factors in early studies [11].

In this article, we describe our experience with the implementation of the first three monoclonal antibody therapies available for use in the treatment of SARS-CoV2 over the course of one year and through two major surges in Southern California. Our main outcome measure was treatment failure, as defined by subsequent ED visits, hospitalizations, and death within 30 days of treatment or referral for treatment. We assessed patient-level factors (such as traditional epidemiologic risk factors and measures of socioeconomic vulnerability) as well as the impact of emerging viral variants on the effectiveness of monoclonal antibody therapy.

## Methods

### Study design and participants

In this retrospective cohort analysis, we examined the records of 2209 UCLA Health patients who were either referred for outpatient Mab therapy for COVID-19 or received Mab therapy in the ED at Ronald Reagan Medical Center and Santa Monica Hospital between December 10, 2020, and December 10, 2021. All data were de-identified during analysis and the UCLA Office of Human Research Protection Program waived the need for informed consent given that this is a retrospective study (IRB #21–00041). The catchment area of UCLA Health largely includes the northern and western portions of LA County and outlying areas, representing one of the most populous and diverse areas of the country. Patients with select risk factors initially were eligible for treatment if they were at least 18 years old, were within seven days of a positive COVID-19 PCR test and symptom onset, and had identifiable risk factors. While our database only formally captured the date of first positive test as opposed to a verified date of symptom onset, all patients who received monoclonal antibody therapy were triaged by a Covid treatment team who confirmed that the patients were within the window of eligibility based on symptom onset and had identifiable risk factors. This was typically done through manual chart abstraction and confirmation with the patient in situations of uncertainty. The EUA subsequently expanded to include pediatric patients between the ages of 12–17 and by March 15 we were including individuals ages 12 and up. Not all patients who received referrals for Mab therapy were ultimately treated, largely due to having a referral outside the seven-day window from the date of test positivity or symptom onset, or due to lack of other identifiable risk factors for severe disease as defined by the National Institute of Health and Centers for Disease Control (S1 Table) [11]. Exclusion of records, which was mostly due to administrative errors, is documented in S1 Fig.

Treated patients predominantly received intravenous bamlanivimab in the beginning of the observational period given the proportionally larger supply, though this was discontinued after March 6, 2021 due to the concern for viral variants which rendered bamlanivimab monotherapy less effective. A minority of patients after this time received combination therapy with bamlanivimab in conjunction with etesevimab. Casirivimab/imdevimab was used throughout the interval observed and was the predominant treatment after March 19, 2021. Treatment was explicitly prioritized to those with higher risk scores in the earlier months due to limited supply, but as the supply became more readily available, all eligible referred patients were able to receive treatment.

Patient-level demographic and clinical data were extracted from the electronic health record into a standardized format for analysis. These data were refreshed every two weeks as patients continued to be referred for treatment, and the results of interim analyses were used to guide institutional decision-making. We capped the inclusion of further records into the study at one year from the beginning of the infusion program. Demographic characteristics of included patients are summarized in Table 1. This study was exempt from IRB review by the UCLA Office of Human Research Protection Program (IRB #21–00041) and complies with the Strengthening Reporting for Observational Studies in Epidemiology (STROBE) guidelines [12].

**Table 1:** Demographic characteristics of included patients grouped by location of treatment. Differences between groups assessed with t-tests or chi-square tests where appropriate.

	Referred Patients w/o Treatment	Treated ED Patients	Treated Outpatients	P-Value
	n = 988	n = 179	n = 1042	
**Age**				< .001
**< = 49**	341 (34.5%)	50 (27.9%)	321 (30.8%)	
**50–64**	314 (31.8%)	39 (21.8%)	350 (33.6%)	
**65–79**	237 (24%)	63 (35.2%)	316 (30.3%)	
**80+**	96 (9.7%)	27 (15.1%)	55 (5.3%)	
**Male**	464 (47%)	82 (45.8%)	517 (49.6%)	0.397
**Race/Ethnicity**				< .001
**Not Hispanic/Latino, White**	467 (47.3%)	70 (39.1%)	513 (49.2%)	
**Hispanic/Latino, All Races**	191 (19.3%)	48 (26.8%)	226 (21.7%)	
**Not Hispanic/Latino, Asian**	48 (4.9%)	15 (8.4%)	67 (6.4%)	
**Not Hispanic/Latino, Black**	69 (7%)	22 (12.3%)	64 (6.1%)	
**Not Hispanic/Latino, Other Race**	92 (9.3%)	20 (11.2%)	82 (7.9%)	
**Unknown Ethnicity or Race**	120 (12.1%)	4 (2.2%)	88 (8.4%)	
**BMI**				< .001
**Normal BMI**	289 (29.3%)	52 (29.1%)	245 (23.5%)	
**Underweight**	19 (1.9%)	7 (3.9%)	15 (1.4%)	
**Overweight**	292 (29.6%)	44 (24.6%)	351 (33.7%)	
**Obese**	276 (27.9%)	43 (24%)	327 (31.4%)	
**Severely Obese**	54 (5.5%)	14 (7.8%)	78 (7.5%)	
**Lung disease**	104 (10.5%)	27 (15.1%)	129 (12.4%)	0.154
**Cardiovascular disease**	91 (9.2%)	17 (9.5%)	92 (8.8%)	0.934
**Kidney disease**	150 (15.2%)	29 (16.2%)	179 (17.2%)	0.475
**Hypertension**	338 (34.2%)	53 (29.6%)	423 (40.6%)	0.001
**Diabetes**	167 (16.9%)	27 (15.1%)	200 (19.2%)	0.244
**Immunocompromised**	8 (0.8%)	3 (1.7%)	7 (0.7%)	0.385
**Vaccination status**				< .001
**Not vaccinated**	626 (63.4%)	146 (81.6%)	503 (48.3%)	
**Fully vaccinated**	332 (33.6%)	33 (18.4%)	515 (49.4%)	
**Partially vaccinated**	30 (3%)	0 (0%)	24 (2.3%)	
**Social Vulnerability Index**				< .001
**Low**	457 (46.3%)	60 (33.5%)	540 (51.8%)	
**Moderate**	284 (28.7%)	49 (27.4%)	267 (25.6%)	
**High**	210 (21.3%)	61 (34.1%)	206 (19.8%)	
**Insurance**				< .001
**Medicare**	268 (27.1%)	78 (43.6%)	276 (26.5%)	
**Medi-Cal**	121 (12.2%)	28 (15.6%)	122 (11.7%)	
**Other Insurance**	580 (58.7%)	64 (35.8%)	628 (60.3%)	
**Uninsured**	4 (0.4%)	0 (0%)	1 (0.1%)	
**COVID-19 Risk Score**				< .001
**Low**	494 (50%)	59 (33%)	457 (43.9%)	
**Moderate**	338 (34.2%)	89 (49.7%)	389 (37.3%)	
**High**	156 (15.8%)	31 (17.3%)	196 (18.8%)	
**Index Date on or After 1 Jun**	592 (59.9%)	70 (39.1%)	726 (69.7%)	< .001
**Treatment**				< .001
**Not treated**	988 (100%)	0 (0%)	0 (0%)	
**Bamlanivimab only**	0 (0%)	92 (51.4%)	214 (20.5%)	
**Bamlanivimab/Etesevimab**	0 (0%)	1 (0.6%)	101 (9.7%)	
**Casirivimab/Imdevimab**	0 (0%)	86 (48%)	727 (69.8%)	
**ED Visit, Admission, or Death w/in 30 days**	64 (6.5%)	37 (20.7%)	39 (3.7%)	< .001
**ED Visit w/in 30 Days**	61 (6.2%)	36 (20.1%)	38 (3.6%)	< .001
**Admission w/in 30 Days**	62 (6.3%)	32 (17.9%)	33 (3.2%)	< .001
**Death w/in 30 Days**	10 (1%)	1 (0.6%)	2 (0.2%)	0.054

### Statistical analysis

The primary outcome of our analysis was the composite 30-day incidence of ED visits, hospitalizations, or death following the index date. The secondary outcomes of our analysis were the 30-day incidence of emergency department visit, hospitalization, or death following the index date, considered separately. The index date was defined as either the date of referral for outpatients or the date of the medication administration for those presenting in the ED (S1 Fig). To estimate the average treatment effect in the treated group (ATT) accounting for major confounders, we used an inverse probability of treatment weighting (IPTW) approach [13, 14]. Propensity scores were estimated using a first-stage logistic regression. Clinically and epidemiologically relevant covariates from the electronic health record preselected by the research team were included in propensity score estimation. These included age, sex, race/ethnicity, body-mass index, comorbidities (lung, heart, or kidney disease; hypertension; diabetes; immunocompromised status), social vulnerability, and insurance coverage. Social vulnerability was quantified using the CDC’s Social Vulnerability Index (SVI), which accounts for demographic, socioeconomic, and built factors in a patient’s community [15]. We used Kaplan-Meier curves and univariate Cox proportional hazards models to assess relationships between individual patient characteristics and our primary and secondary outcomes. Log-rank tests (p < .05) were used to identify factors significantly associated with the primary and secondary outcomes.

After checking for covariate balance following propensity score estimation (S2 Fig), we then used a weighted Cox proportional hazards model to estimate the treatment effect. This model included an interaction term with vaccination status and was stratified by time to account for the changes in characteristics of referred patients over the study period. Proportional hazards assumptions were validated by examining the relationship between the scaled Schoenfeld residuals and time for the treatment variable.

All analysis was conducted using R 4.0.4 software.

### Viral sequencing

Given the implications of emerging variants on treatment failures, we sequenced respiratory samples from a representative sub-group of study patients who were ultimately hospitalized from December 2020 to July 2021. Respiratory samples were sequenced on MiSeq (Illumina, La Jolla, CA) either by amplicon-based ARTIC protocol or shotgun metagenomics approach as described previously [16]. Pangolin COVID-19 Lineage Assigner was used for lineage analysis.

## Results

Of 2209 patients, 988 were referred for treatment but did not receive treatment, 179 received Mabs in the ED and 1042 patients were treated in an outpatient setting. Those treated in the ED were without referral. (Table 1, S1 Fig). A peak in referrals was noted in mid-January and then again in late July. The peak in hospitalizations was significantly greater during the winter surge than it was during the summer surge (S3A–S3C Fig).

For outpatients with both available referral and administration dates, the average time-to-treatment was 1·5 days. Among outpatients, 48·3% were unvaccinated and 45·4% scored moderate to high on the social vulnerability index. Among those who received Mabs in the ED, 63·4% were unvaccinated and 50·0% scored moderate to high on the social vulnerability index (Table 1).

Significant factors associated with the composite outcome included: history of lung disease (HR 7·13; 95% CI 5·12, 9·95), cardiovascular disease (HR 2·61; 95% CI 1·72, 3·95), kidney disease (HR 2·37; 95% CI 1·66, 3·39), and immunocompromised state (HR 6·59; 95% CI 2·91–14·94) as well as high social vulnerability index (HR 2·29, 95% CI 1·56–3·36) (Table 2). Eighty-five respiratory samples from hospitalized patients were reviewed and sequencing data was acquired for 46. The remainder were unable to be sequenced due to low viral load (high cycle thresholds). We identified sixteen total variants, with 39·1% of these patients confirmed to have the Epsilon variant (B.1.427/429), all between the months of December-March. Two Alpha variants (B.1.1.7) were identified between April and May and one delta variant was identified in July.

**Table 2:** Crude Hazard ratios for 30-day risk of ED visit, hospital admission, or death across all treatment groups for selected covariates.

Variable		Events/N	HR (univariable)
**Age**	< = 54	44/931	-
	55–64	21/485	0.92 (0.54–1.54, p = 0.738)
	65+	75/794	2.05 (1.41–2.97, p<0.001)
**Gender**	Female	64/1147	-
	Male	76/1063	1.30 (0.93–1.81, p = 0.124)
**Race/Ethnicity**	Not Hispanic/Latino, White	57/1050	-
	Hispanic/Latino, All Races	46/465	1.85 (1.25–2.72, p = 0.002)
	Not Hispanic/Latino, Asian	9/130	1.28 (0.63–2.58, p = 0.494)
	Not Hispanic/Latino, Black	16/155	1.95 (1.12–3.40, p = 0.018)
	Not Hispanic/Latino, Other Race	9/194	0.85 (0.42–1.72, p = 0.652)
**BMI**	BMI < 35	115/1744	-
	BMI > = 35	23/366	0.95 (0.61–1.49, p = 0.823)
**Lung Disease**	No history	76/1950	-
	Positive history	64/260	7.13 (5.12–9.95, p<0.001)
**Cardiovascular Disease**	No history	112/2010	-
	Positive history	28/200	2.61 (1.72–3.95, p<0.001)
**Hypertension**	No history	71/1396	-
	Positive history	69/814	1.69 (1.21–2.35, p = 0.002)
**Diabetes**	No history	101/1816	-
	Positive history	39/394	1.83 (1.26–2.64, p = 0.001)
**Kidney Disease**	No history	97/1852	-
	Positive history	43/358	2.37 (1.66–3.39, p<0.001)
**Immunocompromised**	No history	134/2192	-
	Positive history	6/18	6.59 (2.91–14.94, p<0.001)
**Vaccine Status**	Not Fully Vaccinated	117/1330	-
	Fully Vaccinated	23/880	0.29 (0.18–0.45, p<0.001)
**Social Vulnerability Index**	Low	52/1057	-
	Moderate	29/601	0.99 (0.63–1.55, p = 0.952)
	High	52/477	2.29 (1.56–3.36, p<0.001)
**Insurance Status**	Other Payor	123/1939	-
	Medi-Cal	17/271	0.99 (0.59–1.64, p = 0.956)
**Index Date**	Before 2021-06-01	87/821	-
	After 2021-06-01	53/1389	0.35 (0.25–0.49, p<0.001)
**Treatment**	Not treated	64/988	-
	Bamlanivimab only	42/306	2.20 (1.49–3.25, p<0.001)
	Combo Therapy (Bam/Ete or Cas/Imd)	34/915	0.57 (0.37–0.86, p = 0.008)

**Fig 1:**
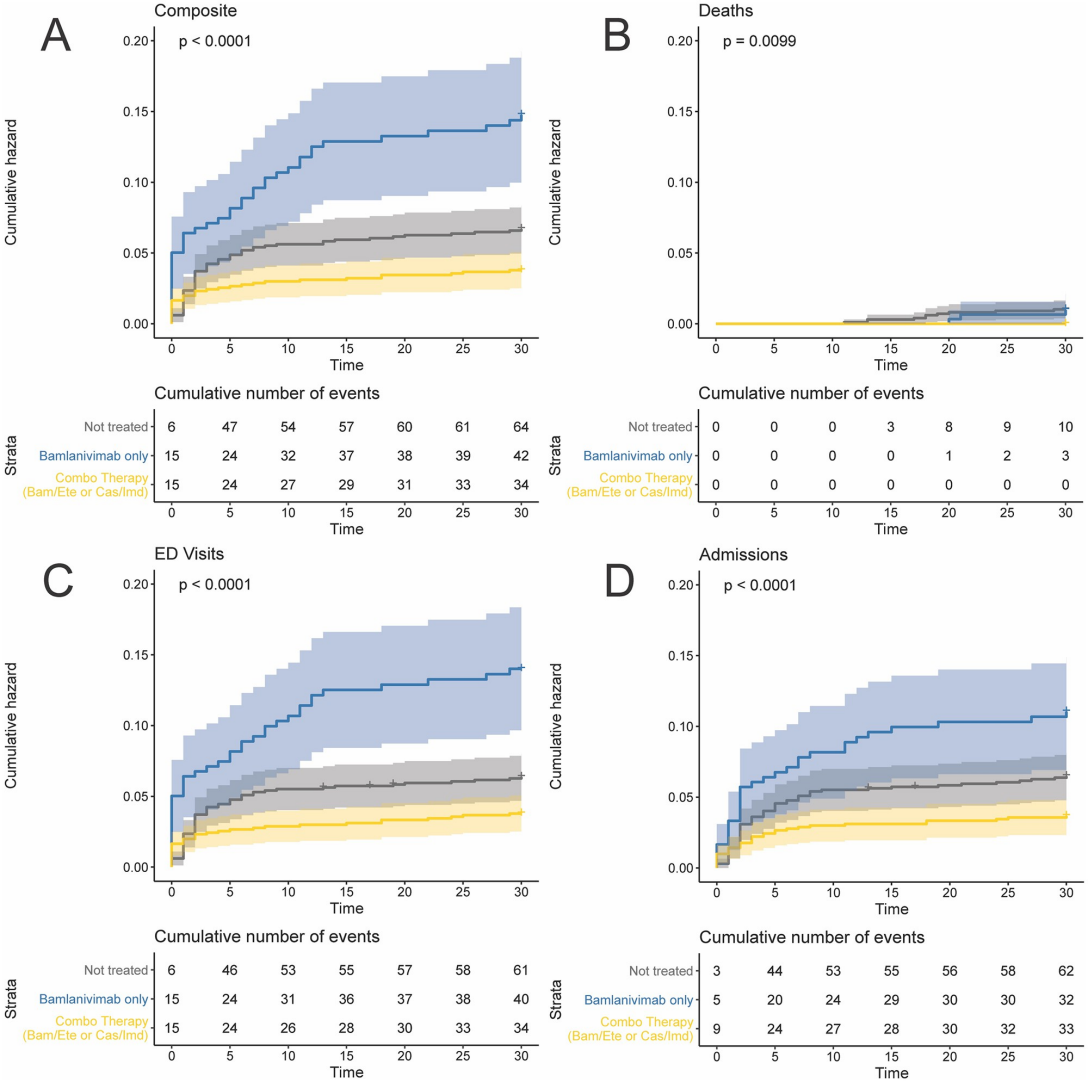
Unadjusted Kaplan-Meier Curves for 30-day Risk of (A) ED Visit, Hospital Admission, or Death; (B) Death Only; (C) ED Visit Only; (D) Hospital Admission Only Stratified by Treatment Status. Shading represents 95% confidence interval. Significant differences between curves are indicated with log-rank p-values.

**Fig 2:**
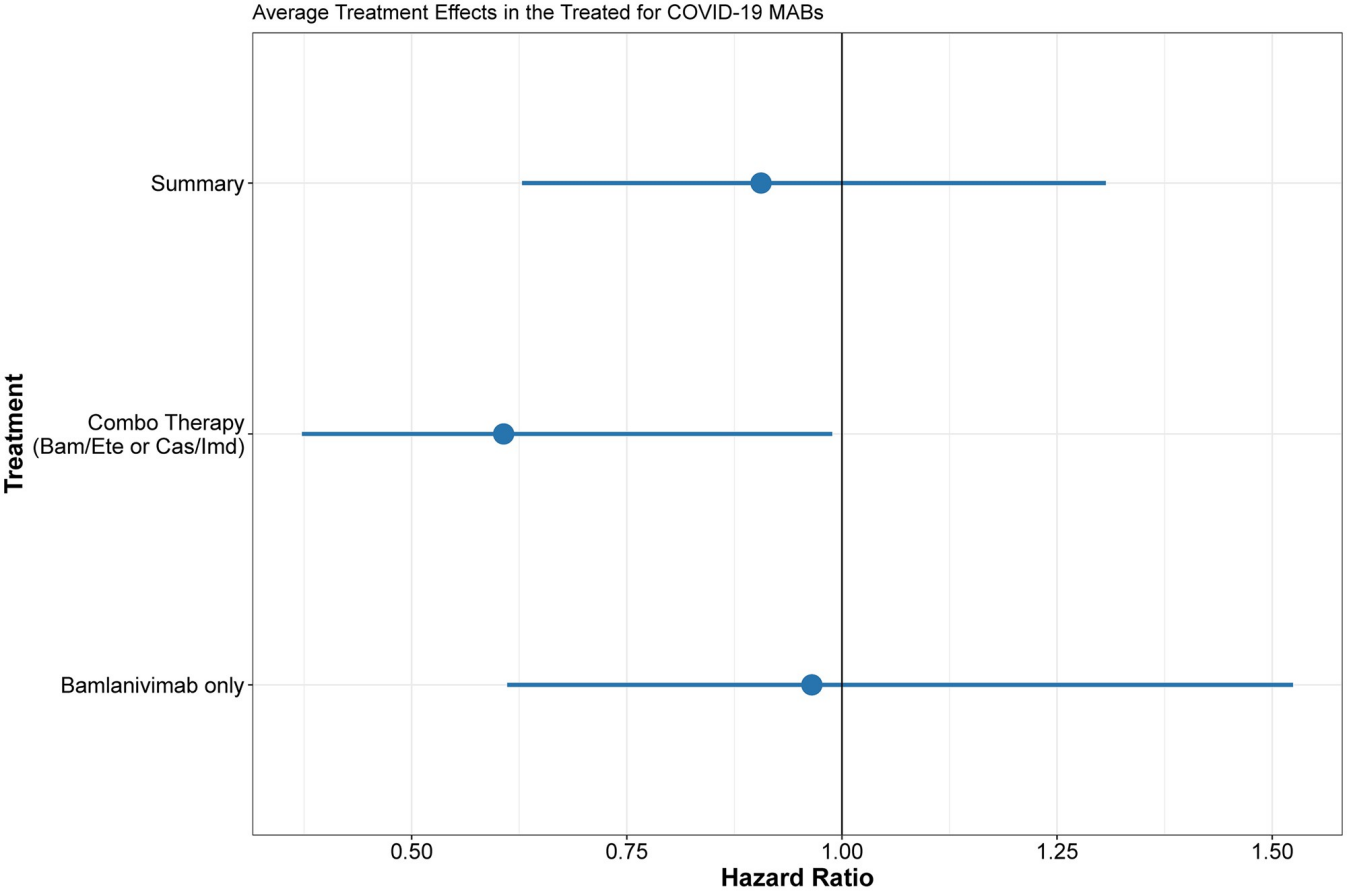
Estimated average treatment effects in the treated (ATT) for pooled, combo therapy only, and bamlanivimab Cox proportional hazards models. Observations in each cox model weighted by estimated propensity score from first stage logistic regression. Error bars represent 95% confidence interval.

Over the entirety of the study period, composite 30-day mortality, ED visits and hospital admissions were significantly reduced in the combination therapy group (bamlanivimab/etesevimab or casirivimab/imdevimab) compared with bamlanivimab or no treatment groups over the entire study (aHR ·60; 95% CI ·37, ·99). (Figs 1 and 2). Subgroup analyses showed that in unvaccinated patients with moderate-to-high risk, the benefit of combination Mab therapy with respect to the primary outcome was even greater (aHR ·38; 95% CI ·20, ·77), while no benefit was evident in fully vaccinated patients with similar risk (aHR 2·31; 95% CI ·71, 7·57).

In the overall study population, the observed benefit of Mab therapy waned over time, as evidenced when considering data from only the second major wave of infections in 2021 (those after June 1^st^, or what has been referred to as the Delta surge), when outcomes were similar between those treated with combination therapy and untreated controls as compared to the first wave (the winter surge of 2020–2021, which in California was due to the Epsilon variant) (S4 and S5 Figs). Decreasing effectiveness of Mabs over time was also observed in patients who were not fully vaccinated, specifically (S6 and S7 Figs), in part due to a reduction in the overall risk of the patients who were treated. Our sequencing data were limited to hospitalized patients does not allow us to conjecture on the impact of variants on treatment effectiveness and outcomes.

## Discussion

Our data show that casirivimab/imdevimab and bamlanivimab/etesevimab monoclonal antibody therapy, but not bamlanivimab alone, when given to high-risk patients with mild-to-moderate COVID-19 symptoms demonstrated effectiveness in reducing 30-day ED visits and hospital admissions. These monoclonal antibody treatments conferred an even greater benefit among patients who were not fully-vaccinated. Prior published data have shown that bamlanivimab monotherapy for ambulatory patients with COVID-19 had demonstrated effectiveness in reducing hospitalizations, even among vaccinated patients [17–21]. In our study, bamlanivimab failed as monotherapy likely to a large extent due to the emergence of the Epsilon variant which was noted in over one-third of hospitalized patients based on sequencing data. Bamlanivimab was removed permanently from distribution by the California Department of Public Health on March 19, 2021 and bamlanivimab/etesevimab was briefly removed from distribution from May 26, 2021 to September 7, 2021 given concerns for the B.1351 and P.1 variants. Those who received bamlanivimab, a drug that was completely ineffective in California, appeared to do worse than those not treated in the unadjusted models, likely because on average those who were treated with any monoclonal were higher risk than those who were not treated at all.

We also note that the effectiveness of casirivimab/imdevimab therapy has decreased over time, with outcomes after June 1^st^ similar across treated and untreated groups. The reasons for this are likely multifactorial, the most significant of which being the changing characteristics of patients referred for treatment. Notably, we observed that referred patients were increasingly vaccinated with lower risk for complications as the year progressed, reflecting the local impact of both broader public health interventions and increased availability of treatment. As higher risk, non-immune patients would have been both the earliest infected and earliest vaccinated, as the year progressed, the remaining susceptible population was at lower risk for complications overall. As corroborated by our subgroup analyses, the marginal benefit of treatment in such a population is limited. Furthermore, the number of hospitalizations overall was significantly lower during the summer surge (due to the Delta variant) compared with the winter surge which was dominated by the Epsilon variant. This again likely was more reflective of improved vaccination status rather than a difference in virulence between the two variants, though we cannot comment for certain in the absence of sequencing data for all patients.

While LA County has a higher COVID-19 vaccination coverage than the national average, massive disparities persist with respect to vaccination and underlying risk across race and class [22]. Indeed, about 20% of our treated patients had a high social vulnerability index and patients with a high social vulnerability index were at a statistically increased risk of ED visits, hospitalizations or death within 30-days. This finding is not surprising considering socially vulnerable populations are at higher risk of poorer outcomes during public health emergencies due to factors such as poverty, food insecurity, household density and composition, or housing type which can all play a role in COVID-19 transmission and medical resource availability [15, 23]. In light of our results, outpatient COVID therapeutics including Mabs still have an important role to play when considering the larger-scale efforts to safeguard the health of the most vulnerable populations in our community.

Our finding that chronic medical conditions such as lung disease, cardiovascular disease and immunocompromised status are associated with poorer outcomes is in line with prior published data [18]. Surprisingly, patients with an increased BMI were not at a significantly increased risk in our study, contrary to what has been published in the past [24]. The effect of vaccination on each of these previously determined high risk groups warrants further study.

A higher proportion of the patients who went to the ED for treatment were admitted within 30-days. This is likely reflective of the fact that patients who visited the ED were likely more ill as they were not being referred there for treatment but rather visiting the ED because of their covid symptoms and received treatment with monoclonal antibodies at the discretion of the ED clinician. Furthermore, they were more likely to have delayed care as over 35% of those treated in the ED had high socioeconomic vulnerability index scores.

We note several limitations with this study. First, we conducted this study within a single health system, affecting its larger generalizability both with respect to the patient population itself and the internal processes by which patients are referred for and ultimately access treatment. Additionally, beyond screening by the outpatient team to determine eligibility for treatment, we were unable to precisely characterize time from symptom onset to treatment from the data we extracted and thus cannot make conclusions regarding expedited or delayed treatment. Similarly, we were unable to capture the specific reasons why patients who were referred but not treated did not receive treatment in our extracted dataset but know this was a combination of not meeting eligibility criteria and personal choice, particularly in cases of relatively mild or improving disease. As with any study that involves a large administrative database, we also experienced some issues with missing or incorrectly entered primary data, including SARS-CoV2 test results, but the data we chose to ultimately analyze were complete in their key features and cross-referenced against manual records as necessary. Finally, while the covariates pre-selected by the research team were strongly hypothesized to be the most relevant to the analysis, residual confounding from both measured and unmeasured variables is still possible.

While vaccinations will be the lynchpin in controlling this pandemic, vaccine hesitancy has emerged as one of the top reasons for lower-than-expected uptake. On January 27, 63% of eligible Americans received the primary series, despite the majority having had access to the vaccines in the eight months prior [25].. As of October 30, 68% of all eligible Americans (and 78% of those 18 and older) received the full primary series. Thus, while a substantial number of people remain unvaccinated, the need for effective outpatient treatment to reduce strain on the health system remains high. Molnupiravir [26] and paxlovid [27], two oral antiviral therapies which have been given EUA, will not replace the benefits of vaccination but have decreased the strain on health systems in areas where they have been effectively scaled.

The number of resources undertaken to implement monoclonal antibody infusion is not insignificant. In our institution, we estimated that the cost of treating 60 patients in the outpatient setting in the first month of implementation was approximately $25,069. This cost included the cost of RN and MA staffing as well as supplies, but it did not include the time spent by the Medical Director of the Antimicrobial Stewardship Program (TV) and the Director of Clinical Operations (JB) in triaging the first 100-plus referrals. We subsequently were able to secure full time administrative support along with a part-time MD (SW) and RN (JG) to triage all referrals. Because our institution had initially received significantly more bamlanivimab than casirivimab/imdevimab, most of these initial resources ultimately went toward a drug that had very little benefit in our communities at the expense of more widely distributing combination therapy which proved to be effective. Policymakers should use this as a note of a caution when considering whether to spend government funds on the purchase and distribution of drugs with limited data to support their use.

As with any antimicrobial, a greater investment towards stewardship is needed to preserve efficacy in the face of changing variants [28], and furthermore conserve resources in times of scarcity. In December 2021, we received limited supply sotrovimab [29], another monoclonal antibody that had been given emergency use authorization and while it was effective against earlier Omicron strains, it soon became in effective with the subsequent emergency of BA.2, BA.4 and BA.5. To date only one monoclonal antibody, bebtelovimab, remains effective and its benefit remains tenuous and there are no clinical trial data published to date.

Novel Covid-19 outpatient therapeutics continue to have a role for the most vulnerable, but scarcity of supplies and resources remain significant. Judicious use of these treatments, and ensuring maximal benefit should continue to be a priority. Similar to the scaling up of vaccination efforts, public education, ongoing data analysis, transparent dissemination of data, and continued policy that ensures equitable distribution of such treatments remains a critical part of our control efforts [30].

## Supporting information

S1 Fig Simplified data processing diagram. Note 3 outpatients had available medication administration times which were used.
(TIF)

S2 Fig Covariate balance in IPTW models. Standardized mean differences in study covariates across treatment groups for the (A) Bamlanivimab-only model and the (B) Combination Therapy model before and after propensity score weighting.
(TIF)

S3 Fig Patient load curves. Weekly Distribution of All Patients Referred for MAB Therapy at UCLA by (A) COVID-19 Risk Score and (B) Vaccination Status; (C) Bi-weekly Distribution of Hospitalizations within 30-days of Referral Among All Patients Referred for MAB Therapy at UCLA.
(TIF)

S4 Fig Unadjusted K-M curves for primary and secondary outcomes prior to June 1, 2021. Unadjusted Kaplan-Meier Curves for 30-day Risk of (A) ED Visit, Hospital Admission, or Death; (B) Death Only; (C) ED Visit Only; (D) Hospital Admission Only Stratified by Treatment Status for Index Dates Prior to June 1, 2021. Shading represents 95% confidence interval. Significant differences between curves are indicated with log-rank p-values.
(TIF)

S5 Fig Unadjusted K-M curves for primary and secondary outcomes on or after June 1, 2021. Unadjusted Kaplan-Meier Curves for 30-day Risk of (A) ED Visit, Hospital Admission, or Death; (B) Death Only; (C) ED Visit Only; (D) Hospital Admission Only Stratified by Treatment Status for Index Dates On or After June 1, 2021. Shading represents 95% confidence interval. Significant differences between curves are indicated with log-rank p-values.
(TIF)

S6 Fig Unadjusted K-M curves for primary outcome stratified by vaccination status and treatment prior to June 1, 2021. Unadjusted Kaplan-Meier Curves for 30-day Risk of ED Visit, Hospital Admission, or Death Stratified by Treatment and Vaccination Status for Index Dates Prior to June 1, 2021. Shading represents 95% confidence interval. Significant differences between curves are indicated with log-rank p-values.
(TIF)

S7 Fig Unadjusted K-M curves for primary outcome stratified by vaccination status and treatment after June 1, 2021. Unadjusted Kaplan-Meier Curves for 30-day Risk of ED Visit, Hospital Admission, or Death Stratified by Treatment and Vaccination Status for Index Dates After June 1, 2021. Shading represents 95% confidence interval. Significant differences between curves are indicated with log-rank p-values.
(TIF)

S1 Table UCLA health scoring system initially used to prioritize MAB allocation among eligible patients based on risk of complications. Eligible patients >18 years old with positive SARS-CoV2 PCR <7 days.
(CSV)
