# Supplementary figures and images for: On variants and vaccines: The effectiveness of Covid-19 monoclonal antibody therapy during two distinct periods in the pandemic
Source: PLoS One. 2022 Dec 1;17(12):e0278394. doi: 10.1371/journal.pone.0278394 (PMC9714735; doi:10.1371/journal.pone.0278394)

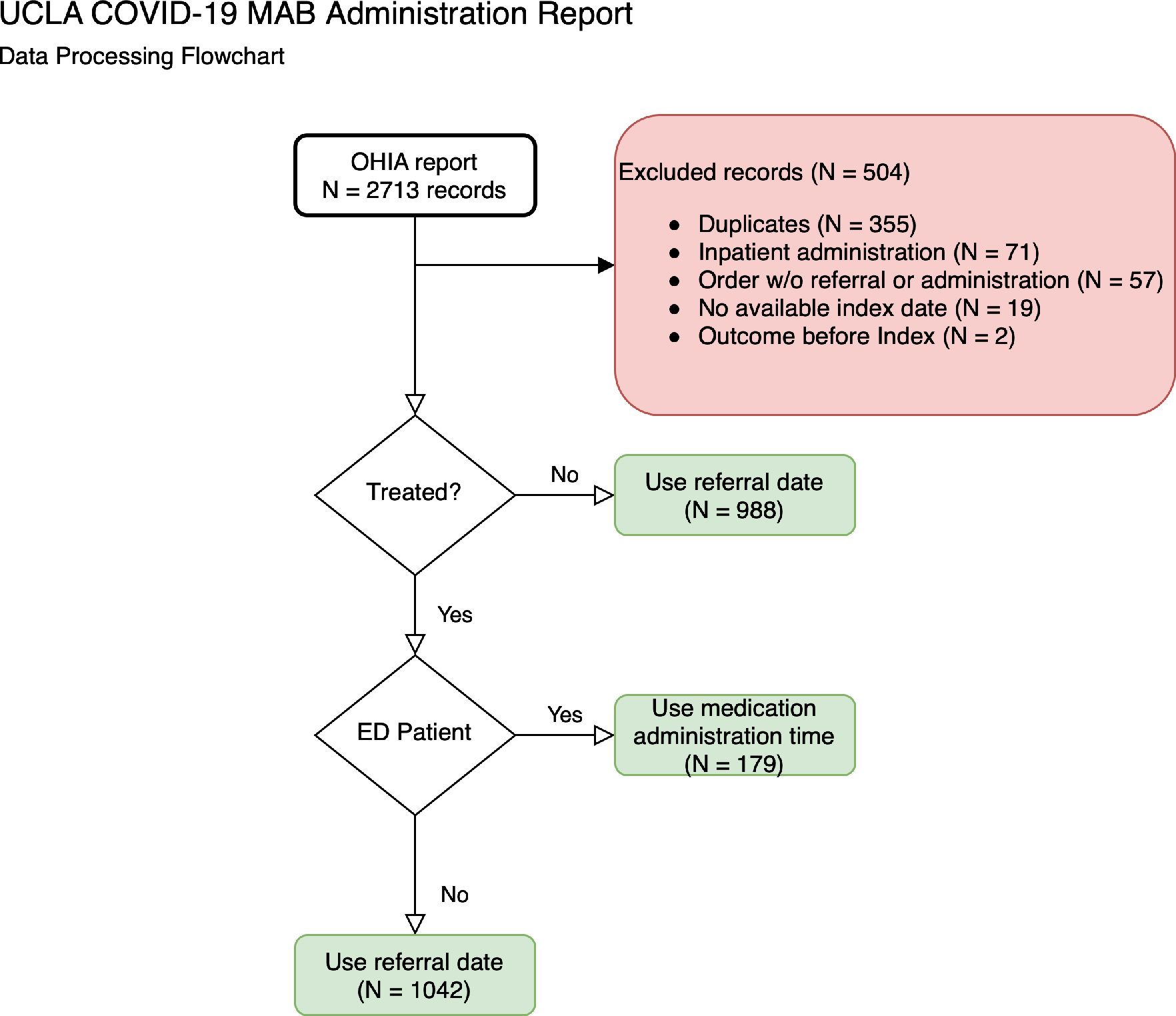

Supplement: S1 Fig — Note 3 outpatients had available medication administration times which were used. (TIF) [file pone.0278394.s001.tif]

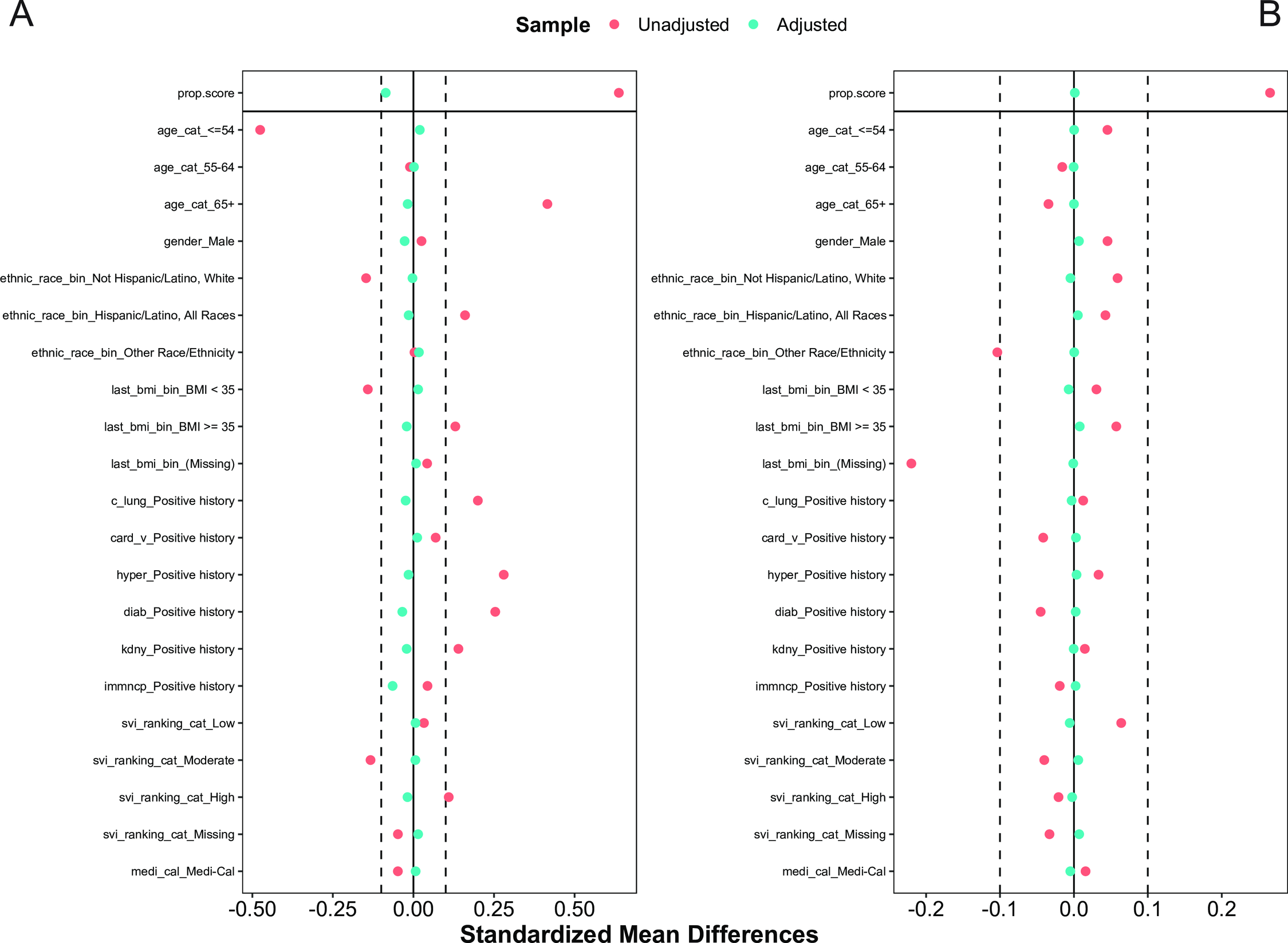

Supplement: S2 Fig — Standardized mean differences in study covariates across treatment groups for the (A) Bamlanivimab-only model and the (B) Combination Therapy model before and after propensity score weighting. (TIF) [file pone.0278394.s002.tif]

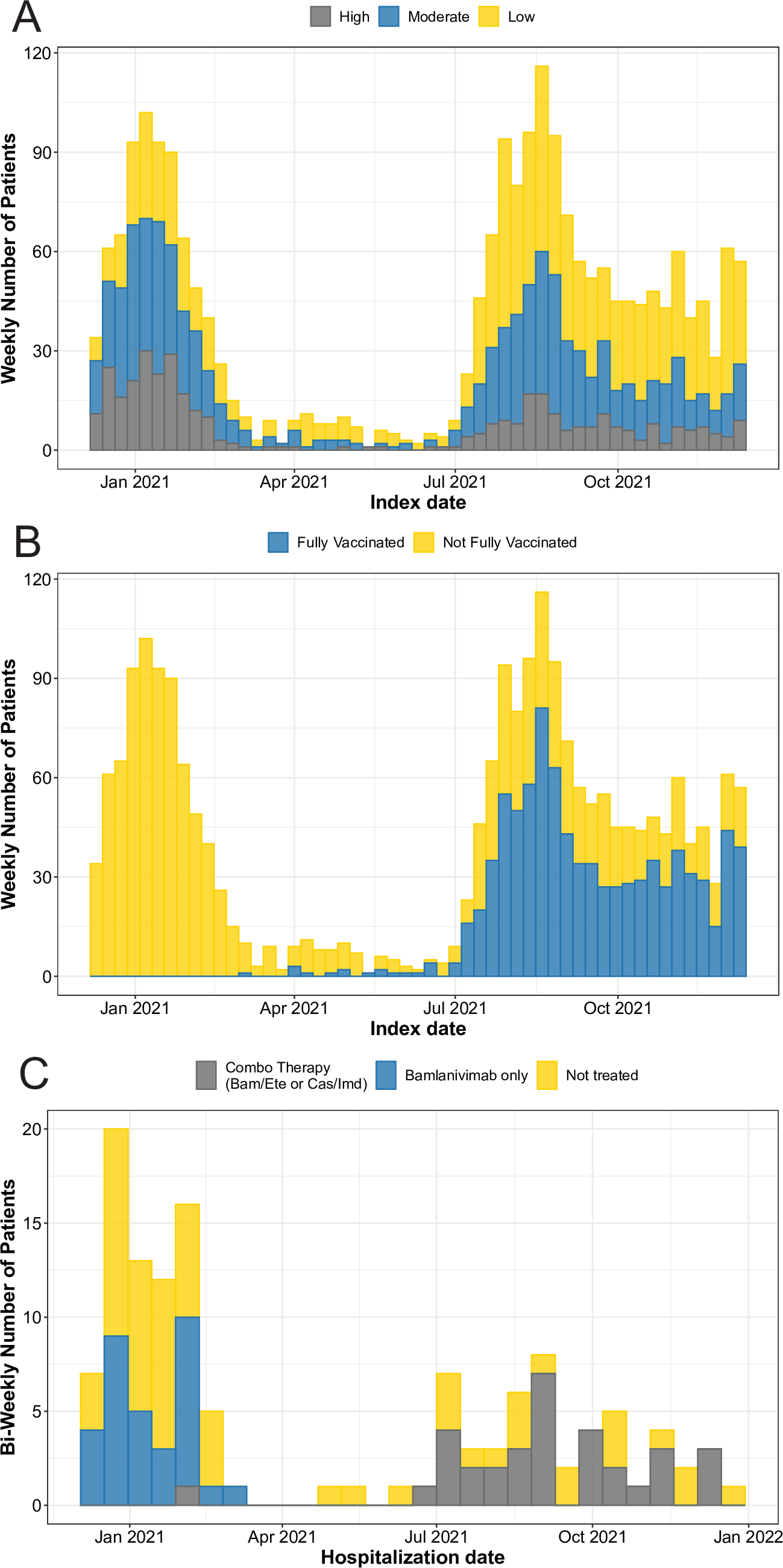

Supplement: S3 Fig — Weekly Distribution of All Patients Referred for MAB Therapy at UCLA by (A) COVID-19 Risk Score and (B) Vaccination Status; (C) Bi-weekly Distribution of Hospitalizations within 30-days of Referral Among All Patients Referred for MAB Therapy at UCLA. (TIF) [file pone.0278394.s003.tif]

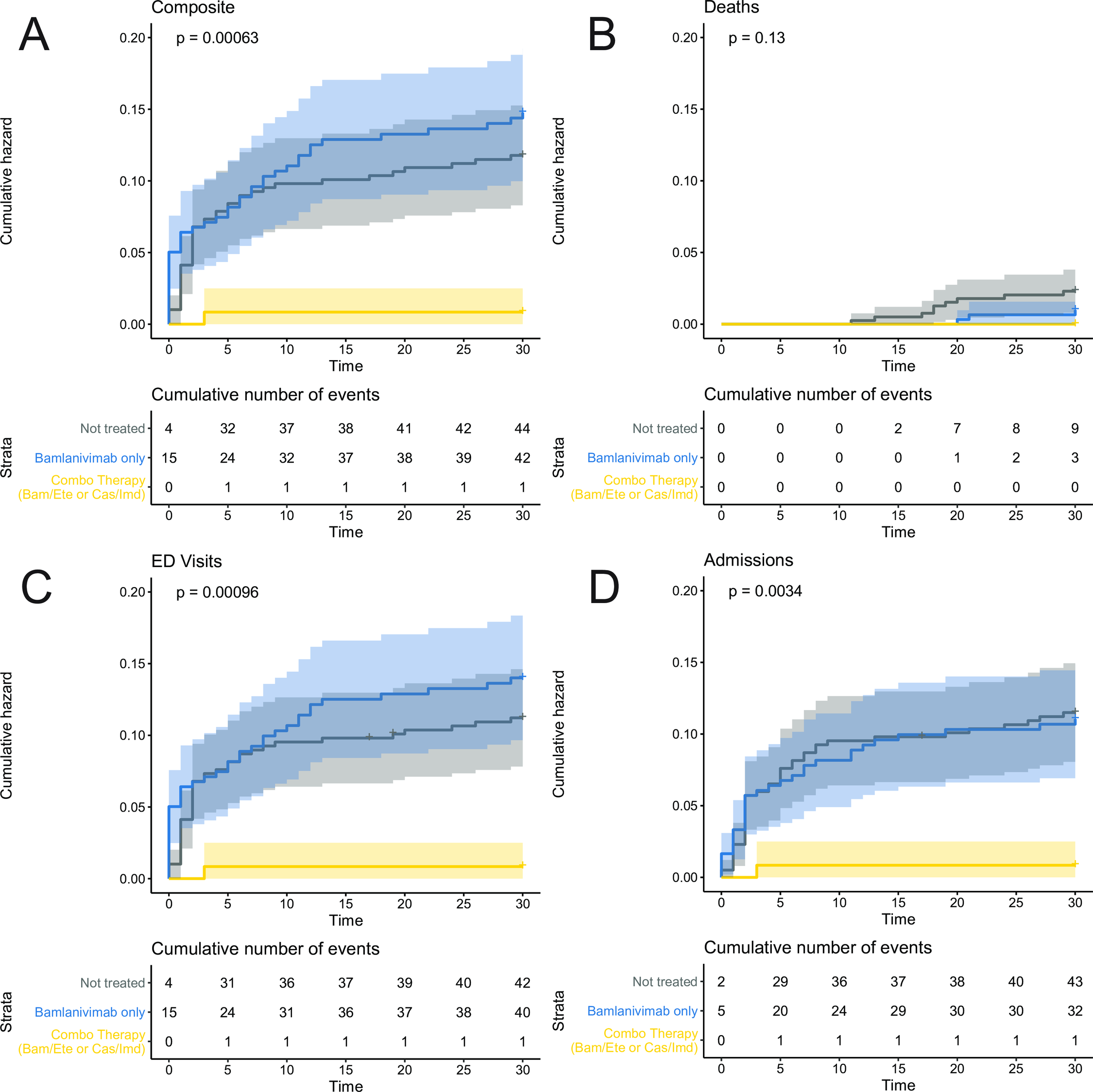

Supplement: S4 Fig — Unadjusted Kaplan-Meier Curves for 30-day Risk of (A) ED Visit, Hospital Admission, or Death; (B) Death Only; (C) ED Visit Only; (D) Hospital Admission Only Stratified by Treatment Status for Index Dates Prior to June 1, 2021. Shading represents 95% confidence interval. Significant differences between curves are indicated with log-rank p-values. (TIF) [file pone.0278394.s004.tif]

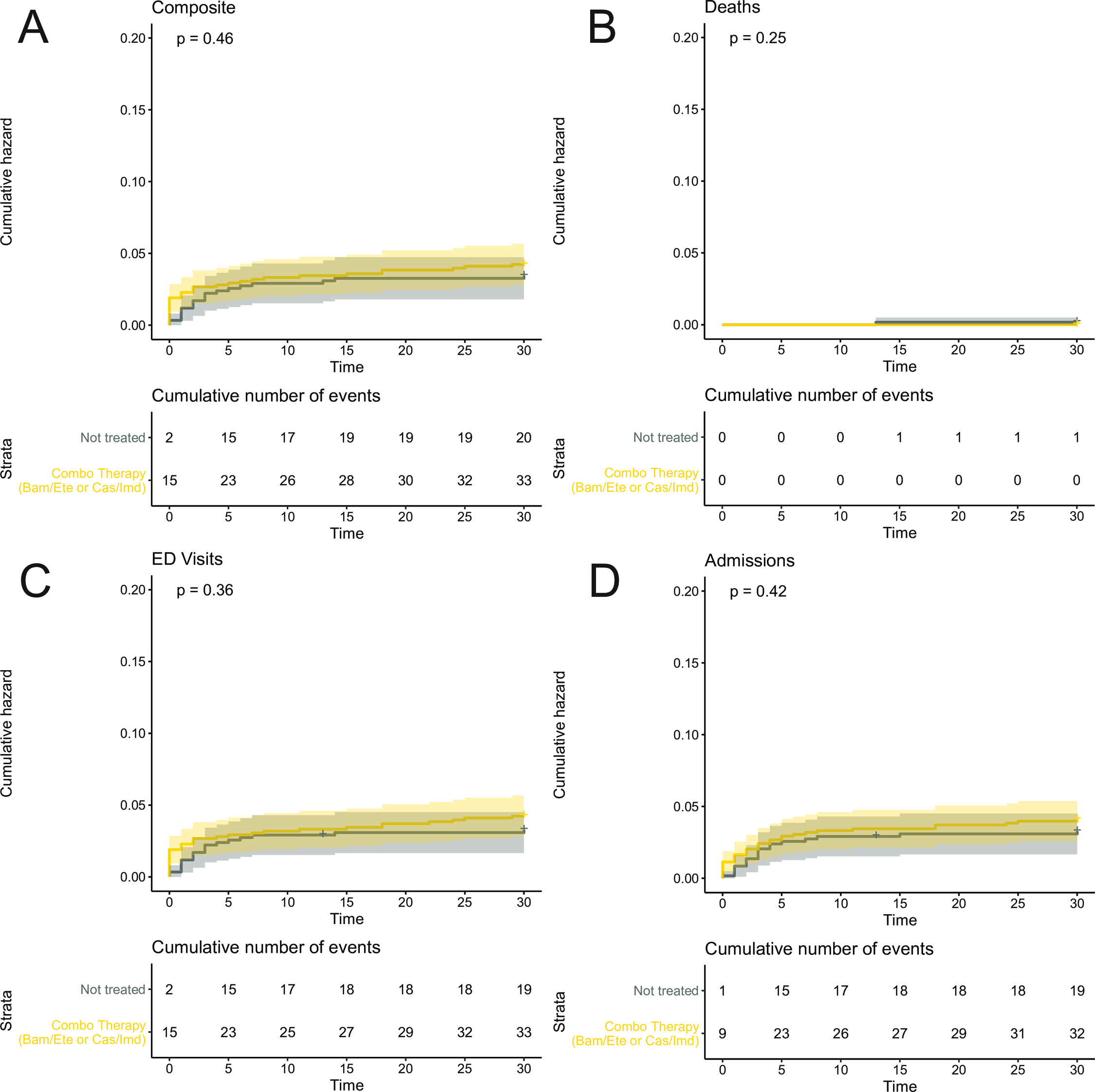

Supplement: S5 Fig — Unadjusted Kaplan-Meier Curves for 30-day Risk of (A) ED Visit, Hospital Admission, or Death; (B) Death Only; (C) ED Visit Only; (D) Hospital Admission Only Stratified by Treatment Status for Index Dates On or After June 1, 2021. Shading represents 95% confidence interval. Significant differences between curves are indicated with log-rank p-values. (TIF) [file pone.0278394.s005.tif]

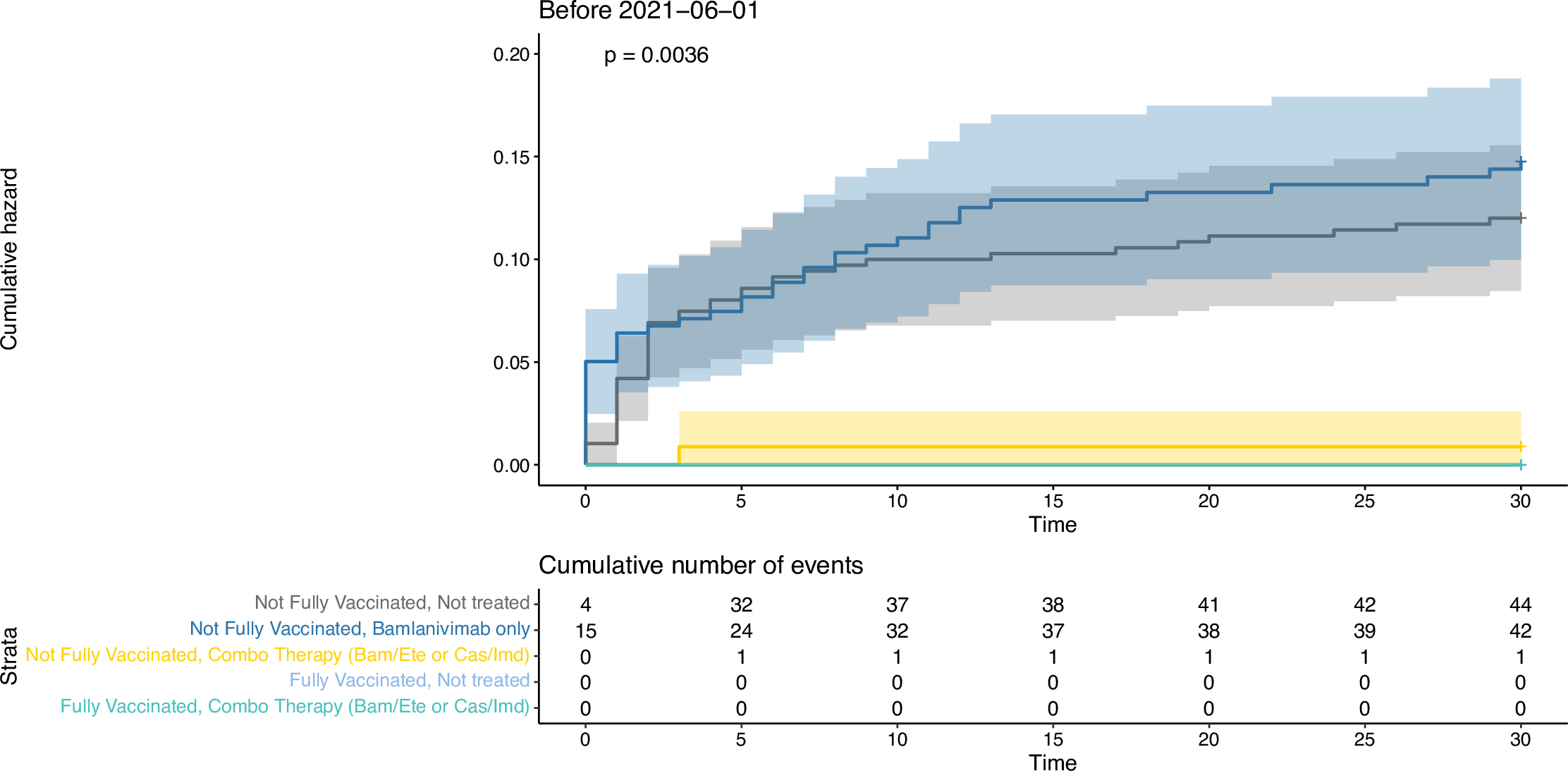

Supplement: S6 Fig — Unadjusted Kaplan-Meier Curves for 30-day Risk of ED Visit, Hospital Admission, or Death Stratified by Treatment and Vaccination Status for Index Dates Prior to June 1, 2021. Shading represents 95% confidence interval. Significant differences between curves are indicated with log-rank p-values. (TIF) [file pone.0278394.s006.tif]

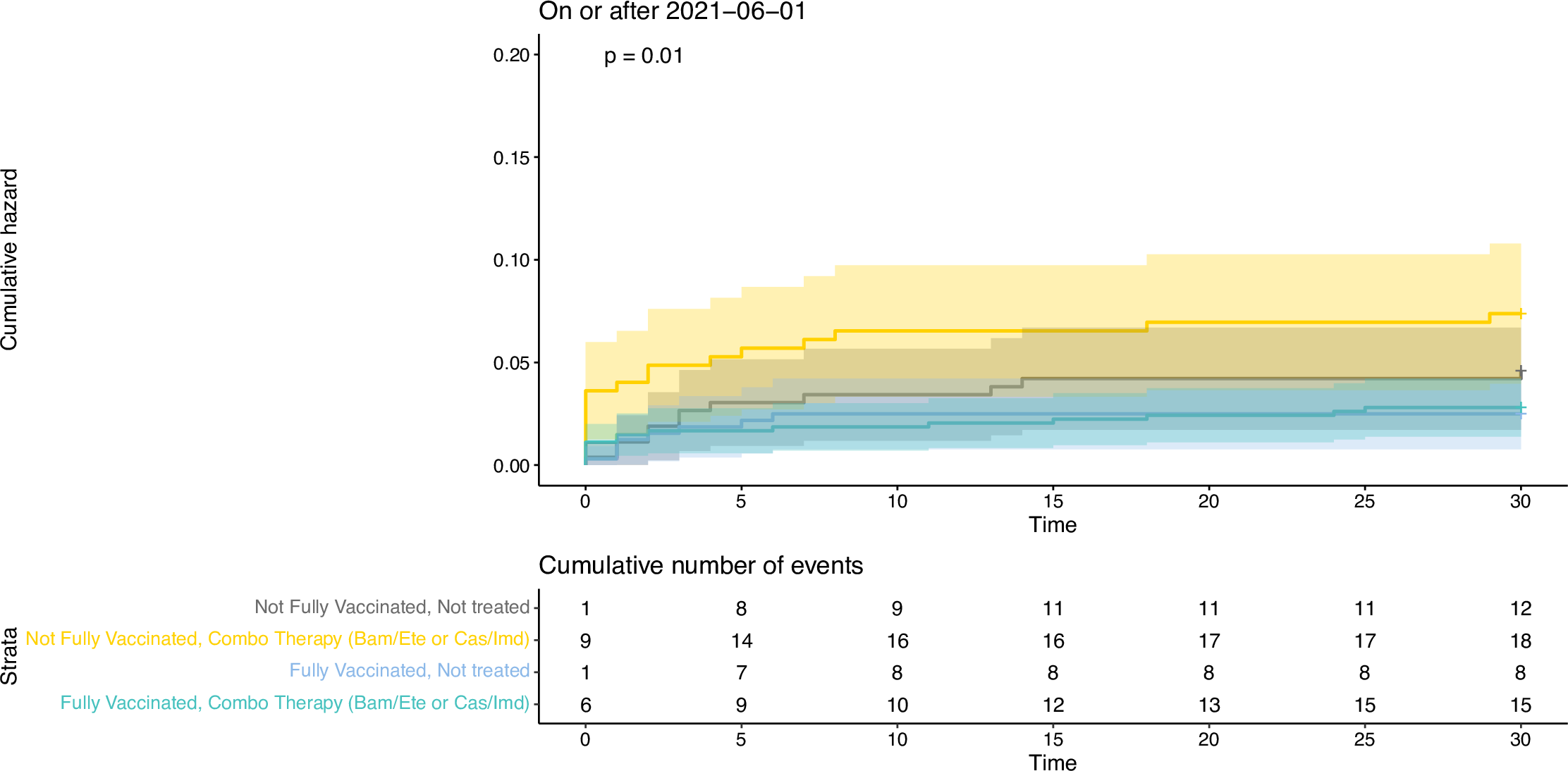

Supplement: S7 Fig — Unadjusted Kaplan-Meier Curves for 30-day Risk of ED Visit, Hospital Admission, or Death Stratified by Treatment and Vaccination Status for Index Dates After June 1, 2021. Shading represents 95% confidence interval. Significant differences between curves are indicated with log-rank p-values. (TIF) [file pone.0278394.s007.tif]
